# Framework of kit-build concept map for automatic diagnosis and its preliminary use

**DOI:** 10.1186/s41039-015-0018-9

**Published:** 2015-10-05

**Authors:** Tsukasa Hirashima, Kazuya Yamasaki, Hiroyuki Fukuda, Hideo Funaoi

**Affiliations:** 1 Learning Engineering Group, Information Engineering, Graduate School of Engineering, 1-4-1, Kagamiyama, Higashi-Hiroshima, Hiroshima, 739-8527 Japan; 2 Faculty of Education, Soka University, Tokyo, Japan

**Keywords:** Concept map, Kit-build, Automatic diagnosis, Goal map, Group map

## Abstract

In this paper, we propose a framework of Kit-Build Concept Map (we call it as KB map) where a concept map made by a learner can be diagnosed automatically. In this research, we have divided the task to make a concept map into two sub-tasks: (1) “segmentation task” where parts of the concept map (nodes and links) are extracted from learning resources and (2) “structuring task” where the extracted parts are integrated into a map. In the framework of kit-build concept map, a learner is given a set of parts of a concept map and then re-builds the concept map by combining the given parts. In this process, the segmentation task becomes a task of recognition of the given parts and the structuring task remains as it is. The concept map should be prepared beforehand by a teacher or domain expert. We call this map “goal map.” The necessary and sufficient parts (kit) are generated by decomposing the goal map. The parts are provided to learners, and then the learners are required to build concept maps (learner maps) by connecting the parts. Since the same parts are used both in the goal map and a learner map, it is possible to find defects in the learner map as the differences from the goal map. By overlaying several learners’ maps, then, a group map can be generated. By comparing the group map with the goal map, differences between the goal map and the group of the learners are detected. We have also realized procedure to re-examine the goal map based on the differences between the group map and the goal map. We have already developed a system that realized the framework of the KB map. Through a preliminary use of the system, we have confirmed that the system works along with the framework of KB map. Evaluation of learning effect is our future work.

## Background

Concept map is a useful tool to promote learners to describe their knowledge or understandings by themselves (Novak & Gowin, 1984). From the viewpoint of teaching, the concept maps built by learners are promising products to examine the students’ understandings (Barenholz & Tamir, 1992; Ozdemir, 2005). Diagnosis of concept maps built by learners, however, remains as a big issue to realize educational interaction through the concept map. A learner sometimes fails to build an adequate concept map, and then it is usually difficult for the learner to be aware of the incompleteness. Therefore, it is necessary to support the learner to find and correct the errors in their maps. Here, educational interaction is composed of two activities: (1) learners give the concept maps to a teacher and (2) the teacher helps the learners to improve their maps. Then, diagnosis means that to examine the learner maps and to find their errors. In a usual classroom, however, it is very difficult for a teacher to examine each concept map built by students in the class in real time.

From the viewpoint of technology-enhanced learning, several investigations have already addressed the automatic diagnosis of learners’ concept maps and provision of appropriate feedback (Chang, Sung, and Chen, 2001; Cimolino and Kay, 2002; Conlon, 2004; Gouli, Gogoulou, Papanikolaou, and Grigoriadou, 2005). Their basic approach to realizing the assessment is to compare the learner’s and the teacher’s concept maps. Some of the investigations have addressed the automatic assessment and paid special attention to handle the cases where learners have misspelled a concept or they have used a synonym or a concept related to the appropriate one, based on natural language processing techniques. Betty’s Brain (Leelawong and Biswas, 2008) has the ability to simulate a concept map built by a learner, and the results of simulation promote the learner to improve the map. Although this research has realized advance diagnosis abilities of concept maps, it is necessary to prepare knowledge base or simulation function for a subject domain. Such preparation is usually impossible for usual teachers. Moreover, a map should be described strictly to keep predefined notation in order to interpret the meaning automatically. It is not easy for teachers and learners to master the notation itself.

As another approach to realizing automatic diagnosis of a concept map built by a learner, there is “closed-end” approach (Taricani and Clariana, 2006). In this approach, a learner is provided to nodes and links that are composed of teacher’s concept map, and the learner is required to make a concept map by combining them (Chang et al., 2001; Novak and Cañas, 2008). Because the learner map is composed of the same elements with the teacher’s map, it is possible to detect the differences between them. As a learner model and its diagnosis, they are shallow ones because learner is able to make a map in the limitation of provided parts. Therefore, they deal with only recall and understand levels in Bloom’s taxonomy (Bloom, Engelhart, Furst, Hill, and Krathwohl, 1956). Besides, the accuracy of the learner model is lower than the case where the elements are drawn by the learner. However, because of the benefit of automatic diagnosis, this approach is also adopted by several investigations.

The task to build a concept map is able to be divided into two sub-tasks: (1) “segmentation task” where parts (that is, nodes and links) of a concept map are extracted and (2) “structuring task” where the extracted parts are integrated into a map (Yamasaki, Fukuda, Hirashima, and Funaoi, 2010). From this point of view, in the closed-end approach, the segmentation task is replaced with recognition task of parts and the structuring task remains as it is. Therefore, a learner is relieved from a burden of the segmentation task and is able to concentrate on structuring task. Similar framework, where the segmentation task is simplified and the structuring task is emphasized, has also been proposed in investigations of “note-taking” (Armbruster, 2000; Kiewra, 1991) or “learning by problem-posing” (Hirashima, Yamasaki, Fukuda, and Funaoi, 2007). In several researches about “note-taking from lecture” (Armbruster, 2000; Kiewra, 1991), the process is categorized into (1) selection process and (2) connection process. In the selection process, information that should be noted are selected by a learner, and in connection process, selected information is connected with each other to create a coherent structure. These researches, then, indicated that the learner’s performance in the connection process has stronger influence to the learner’s comprehension of a lecture than the performance in the selection process. Moreover, if a learner failed to select necessary information in a lecture, it is impossible for the learner to recover the failure. Based on these considerations, Armbruster suggested that to reduce the learner’s load in selection process and to let the learner to focus on connection process are promising to improve the learner’s comprehension of a lecture (Armbruster, 2009).

In our previous investigation of learning by problem-posing in arithmetical word problems, it has been proposed a framework of problem-posing as sentence integration (Hirashima et al., 2007). In this framework, a learner is provided several sentence cards and is required to make word problems by combining the sentence cards. This means that a learner relieves the burden of generation of sentences by him/herself and is able to concentrate on combining the sentences to make an adequate structure of a problem. This approach also simplifies the segmentation task and focuses on the structuring task.

These investigations pay special attention to learning effect of the structuring task and provide a learner with a set of parts of a structure in order to make a learner focus on the structuring task. Based on these considerations about diagnosis of the concept map and learning effect of the structuring task, we have also adopted the closed-end approach of the concept map. We call our framework of the concept map “Kit-Build Concept Map” or “KB map.” We use this term in this paper. A map prepared by teachers or domain experts are called “goal map,” and a map built by a learner is called “learner map.” A set of parts composing the map is called “kit”.

The framework of KB map has the following two characteristics, (A) concept map building task is divided into segmentation task and construction task, and then the segmentation task is replaced by recognition task of parts of a concept map that is “kit,” and (B) a goal map should be prepared as an ideal map that a learner is required to build; the applicable targets of the KB map are restricted and it requires several additional functions for the learning environment. Therefore, it is necessary to propose an adequate way to use KB map under these restrictions.

If we use a concept map as a tool for confirmation and reflection of learning or teaching that has already been carried out with a clear learning/teaching goal, it is possible to adopt the KB map. If a goal concept map could be specified, the map would represent something what learners should acquire through the learning or teaching. In such case, the parts of the goal map should appear in the learning/teaching process explicitly. Therefore, recognition of them is a worthwhile activity in the viewpoints of both learning itself and measuring the results of the learning. Since this is not a special situation in learning/teaching, we believe that the applicable range of the KB map is wide enough. Based on these considerations, preparation of learning material and a goal map is an indispensable phase in the framework of the KB map.

Preparing an adequate goal map is, however, not an easy task even for teachers. Causes of the incompleteness might include not only in the goal map itself but also in learning material or instruction activities. For example, the instruction or learning material often includes unclear or misleading explanation. Moreover, recognition of parts would be influenced by the learner’s prior knowledge that was not included in the learning material. As a solution against this issue, we have been investigating interactive adjustment of a goal map. In this adjustment phase, the system gathers a set of learner maps and then makes a group map by overlaying the learner maps (we call this map “group map”). Since the group map is a kind of representation of understanding of the group of learners, the differences between the group map and the original goal map show the characteristics of the understanding of the group. If there is a link that many learners connected in the same way in the group map and the link is connected in different way in the goal map, a teacher should pay special attention to the link because there might be a common reason for them. By examining the link, related nodes, corresponding learning material, and teaching activity for the material, a teacher might find that the link would be reasonable one. When the link is judged as acceptable one, though it is different from the goal map, the goal map should be adjusted to accept the link. Because this adjustment also leads to additional explanation related to the difference, the adjustment phase of the goal map is also indispensable in KB map. We call this phase “goal map modification.”

In the next section, the framework of KB map is explained as a flow of map building composed of (1) goal map building and generation of a set of parts, (2) learner map building, (3) map diagnosis and goal map modification, and (4) learner map modification. In the “Implementation of KB map system” section, implementation of KB map system is introduced. In the “Preliminary use” section, a preliminary use of the KB map system is reported. The purpose of this use is to examine whether the system can be used along the framework of the KB map, as a previous step to use it in practical learning context. Therefore, this preliminary use is not in a real learning context. Although the above mentioned four steps were conducted, the subjects had made their learner maps while referring to a resource document of the map and their learning results had not been evaluated. Therefore, evaluation of learning effect of activities with KB map system is our important future work. The procedure of this use and its results are explained in the “Preliminary use” section.

## Practical flow of KB map building

**Fig. 1:**
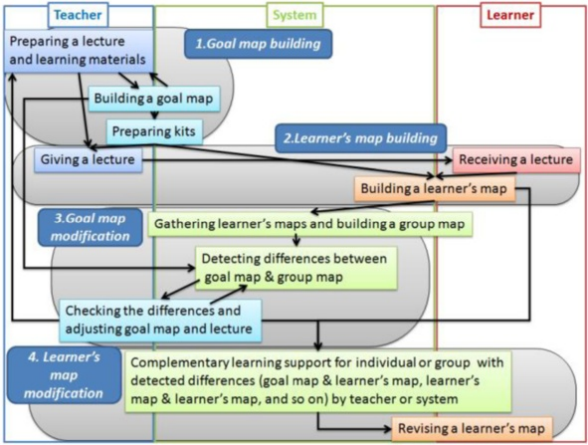
Practical flow of kit-build concept map building

To satisfy the applicable conditions of KB map framework, we have designed a practical flow to build the KB map shown in Fig. 1. In the flow, a teacher and learners are able to interact with each other through concept maps. There are four main phases: (1) goal map building and generation of a set of parts, (2) learner map building, (3) map diagnosis and goal map modification, and (4) learner map modification. Following this flow, each phase is explained in this section. An implementation of this framework is described in the “Implementation of KB map system” section.

**Fig. 2:**
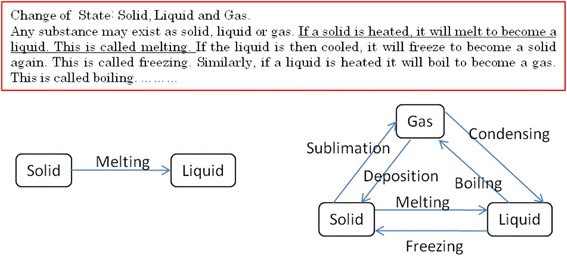
An example of goal map building

**Fig. 3:**
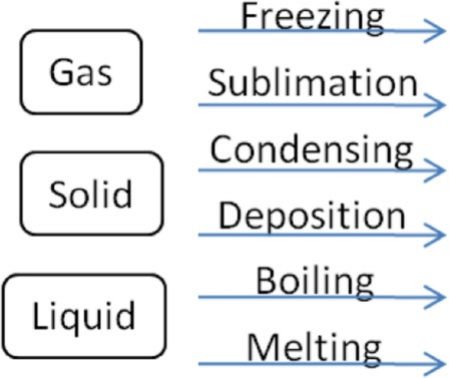
Kit of the goal map

**Fig. 4:**
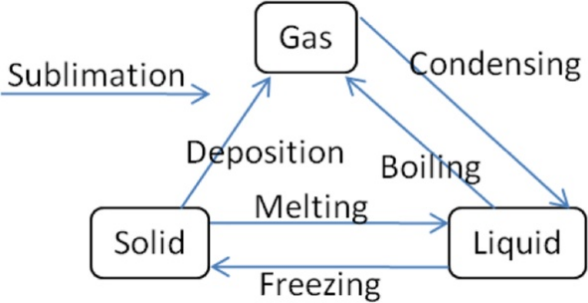
A learner map

**Fig. 5:**
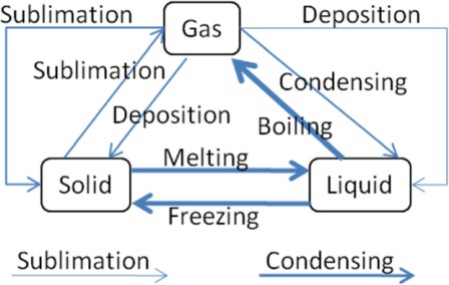
A group map

(1) Goal Map Building: In the process of the goal map building, an author of the goal map is required to input a learning material at first. The teaching or learning should be carried out following the learning material and everything that learners are required to represent in their concept maps should appear explicitly in the material. In other words, the goal map should be composed of the words that explicitly appear in the material. To make a unit of a goal map, an author selects words from a part of a learning material and makes a proposition composed of the selected words. For an example, on the upper side of Fig. 2, there is a part of explanation of “change of state: solid, liquid and gas” in basic science education. In this figure, the author selected a part of the explanation marked by underline. Then, three words (Solid, Liquid, and Melting) were picked up and made a proposition that is composed of two nodes and one link, shown on left lower side. By connecting with several propositions extracted from a learning material, a goal map is completed. A goal map for the explanation of “change of state” is shown on the right lower side of Fig. 2. By decomposing the goal map, a set of parts, that is “kit,” is prepared, as shown in Fig. 3. In our current framework, we have not considered the details of the way to build the first goal map yet. Collaborative building of the first goal map with several experts or teachers is a conventional and promising way (Hirashima et al., 2011). It is necessary for us to sophisticate this goal map building phase as an important future work. On more important idea in goal map building is distractors of nodes or links. Currently, kit is composed of only correct nodes and links. However, a teacher who can build the goal map may prepare useful distractors in addition to the correct ones. Such distractors would contribute to learning by building maps. This is also an important future work.

(2) Learner Map Building: In this framework, learners are supposed to learn the topic represented as the goal map from the learning material and/or the teaching based on the material. Then, as a kind of comprehension test, learner composes a concept map by using provided parts. Figure 4 shows an example of learner map that is not completed. Because a map is composed by connecting links between nodes, all errors are detected as mistakes in link connection. In Fig. 4, “Sublimation” is not used in the learner map. We call this error as “leaving link.” Since “Deposition” link from “Solid” node to “Gas” node in the learner map does not exist in the goal map, this error is called as “excessive link.” Conversely, “Sublimation” link from “Solid” node to “Gas” node and “Deposition” link from “Gas” node to “Solid” node are lacking in the learner map. We call them as “lacking link.” Diagnosis of learner map is explained further in the next subsection.

(3) Group Map Building and Modification of Goal Map: Just after the learner’s map building phase, the learner maps are gathered online and a group map is generated by overlaying them. Figure 5 shows an example of the group map. A link included in a lot of learner maps is drawn by a bold link, and a link included in few learner maps is drawn by a thin link. For example, since “Melting” link from “Solid” node to “Liquid” node is included in almost all learner maps, it is drawn as a bold link. Since “Deposition” link from “Solid” node to “Gas” node is included only in few learner maps, it is drawn as a thin link. Somewhat, bold “Condensing” link disconnected to the group map means that the link is not used in several learner maps.

**Fig. 6:**
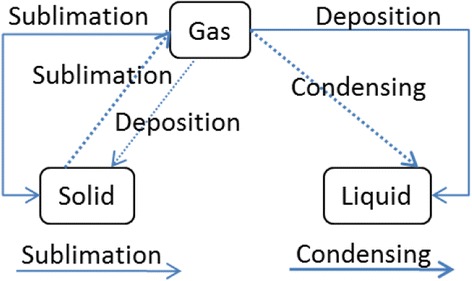
A group-goal difference map

By comparing the group map with the goal map, the difference between ideal understanding and learners’ current understanding is extracted. The differences are represented as “group-goal difference map” shown in Fig. 6. In the group-goal difference map, broken links are lacking ones and solid links are excessive ones. Links that are not connected to the map are leaving links. The group-goal difference map shows that all learners correctly linked “Melting,” “Freezing,” and “Boiling” because these links are not in the map. Then, the map shows that some of the learners failed to link “Sublimation,” “Deposition,” and “Condensing” correctly. In solid links connecting nodes in the map, “Sublimation” and “Condensing” links are bolder than “Deposition” link. This means that more learners could not connect “Sublimation” and “Condensing” links correctly. Therefore, these links are more important in the goal map modification phase. A difference map can be generated in comparison between group map and individual learner map or in mutual comparison of learner maps. Those kinds of difference maps would also provide useful information to support learners, especially in the context of collaborative learning.

If a teacher finds a portion in the goal map where modification is necessary, he/she is allowed to modify the portion in the goal map modification phase. If the teacher finds unclear description in the related learning material, he/she is expected to revise the description. In this example, although the goal map and description themselves are acceptable one, “Sublimation” from “Gas” to “Solid” should be considered in the modification. Several learners used “Sublimation” instead of “Deposition,” while the direct change from “Gas” to “Solid” is explained as “Deposition” in the learning material. Several textbooks have used the term “Sublimation” as direct changes of both directions between “Solid” and “Gas”. Therefore, it would often be acceptable as the “Sublimation” link. In KB map, a teacher is allowed to add alternative link to the goal map. In this case, if “Sublimation” link is added to the goal map as an “alternative link,” then the link in a learner map is not dealt with an error. As for the learning, it would be better to add supplementary explanation about “Sublimation” and “Deposition.” In the implemented system of KB map, the overlaid degree of each link is calculated by counting the number of maps including the same link. Then, the overlaid degree of the link in the group map or difference map is visualized as the thickness of the link as shown in Fig. 5.

(4) Learner Map Modification: After confirmation or modification of the goal map and the learning material, the differences between the goal map and the group map or learner maps are informed to the learners to promote the correction of the learner maps in the phase of learner map modification. For example, with the group-goal difference map shown in Fig. 6, it is easy to point out that the relation between “Gas” and “Solid” were difficult for learners to understand, and the teacher could request learners to read again the corresponding part in the learning material. As for individual feedback, the difference between the goal map and the learner map is shown to a learner. In this feedback, in addition to the excess link and the lacking link, a leaving link that is not connected to the learner map is also pointed out.

## Implementation of KB map system

**Fig. 7:**
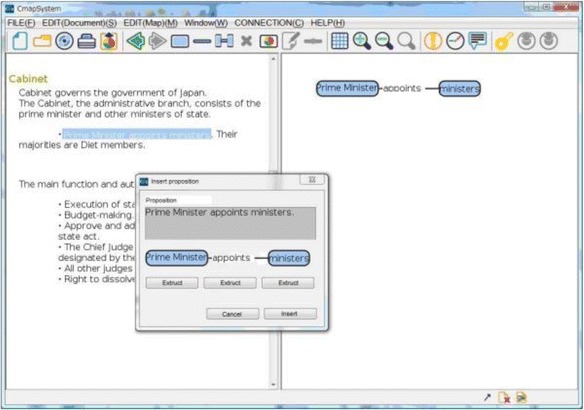
Goal map editor

**Fig. 8:**
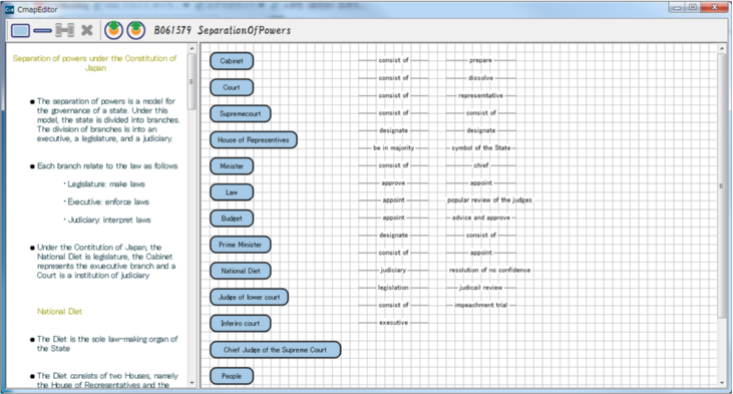
Learner map builder

**Fig. 9:**
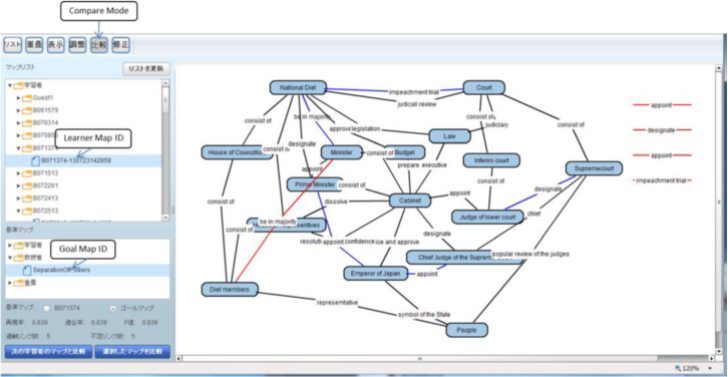
KBmap analyzer

We have already developed a system based on the framework explained in the previous section. This system is called as “KB map System.” It is a web application with three client systems: “Goal Map Editor,” “Learner Map Builder,” and “KBmap Analyzer” and a server system: “KBmap DB.” Goal Map Editor provides a teacher with an environment for a teacher to edit the goal map and confirm a set of parts made from the goal map. Learner Map Builder is an environment where a learner builds a map with the parts. Goal Map Editor and Learner Map Builder have been implemented in Java. KBmap Analyzer has functions to gather learner maps online, to generate a group map, to adjust a goal map, and to visualize the differences between several kinds of maps. This analyzer has been implemented in Flash. KBmap DB has a function to store and share the maps. This system has been developed in Ruby on Rails and MySQL. Interfaces of Goal Map Editor, Learner Map Builder, and KBmap Analyzer are shown in the following subsections as Figs. 7, 8, and 9, respectively (since only Japanese version has been implemented, words in the figures are translated into English). The three client systems are explained in this subsection. Preliminary use of KB map System is reported in the “Preliminary use” section.

### Goal map editor

**Fig. 10:**
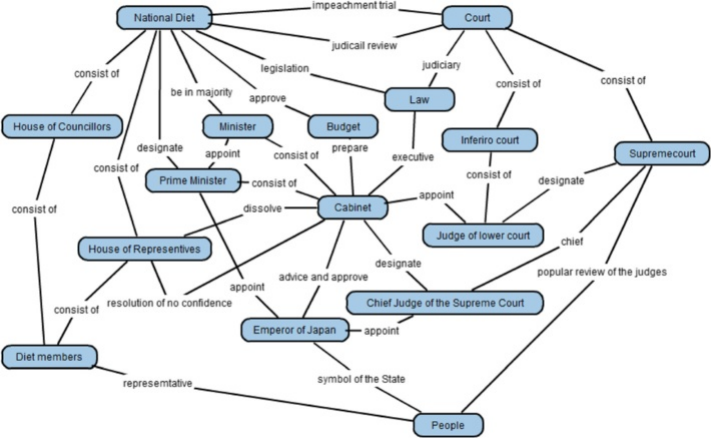
An example of goal map

A teacher is required to make a goal map as an ideal result of learning with a learning material. In Goal Map Editor as shown in Fig. 7, a part of a text of a learning material is shown in the left side. A teacher makes a goal map with words in the text. In this example shown in Fig. 7, a sentence, that is, “Prime Minister appoints ministers” is selected and highlighted. Then, the sentence appeared in the small window in the center of the figure. This is a window to make a proposition from a sentence. In the window, an author of the goal map is required to pick up labels of two nodes and one link from the sentence. In this case, “Prime Minister” and “minister” are selected as the node labels and “appoint” is selected as the link label. A proposition composed of these nodes and link is shown on the right side of Fig. 7. Because parts of generated propositions are extracted from the same material, the meaning of the same label is guaranteed to be the same. By such integration, a map is composed from several propositions that are made from the same learning material. If the teacher accepts the map as the goal map, the map is decomposed and a set of parts of the goal map is generated. Figure 10 shows an example of a goal map. Figure 8 shows an example of a set of parts of the goal map that is given in Learner Map Builder. This goal map and set of the parts were actually used in the preliminary use described in Section Preliminary use.

### Learner map builder

**Fig. 11:**
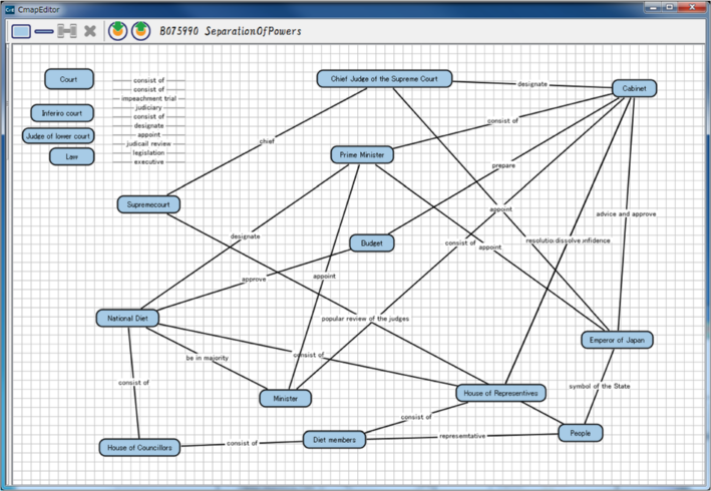
An example of learner map

In Learner Map Builder, a learner firstly downloads a set of parts, that is, nodes and links, as shown in Fig. 8. Then, the learner is required to connect them to build a map that represents his/her understanding. The connection can be carried out by drag and drop operation. When the learner pushes the upload button, the map is sent to KBmap Analyzer. The learner map shown in Fig. 11 is not completed yet because there are several nodes and links that are not connected.

### KBmap analyzer: Learner map

In KBmap Analyzer, learner maps are gathered and analyzed. Each learner map can be observed in the same spatial placement of nodes and links that a learner made. Then, a learner map can be compared with the goal map or another learner map. In the left column of Fig. 9, a learner map and the goal map are selected as the objects of the comparison, and the result is shown in the right side. We call this map “learner-goal difference map.” The spatial placement of nodes and links in the learner-goal difference map can be adopted from either the learner map or the goal map. In this case, spatial placement of the goal map is adopted.

Colored lines are differences between the learner map and the goal map. A lacking link is colored blue and excessive one red. Because a leaving link does not appear in the goal map, it is also colored red in the same with the excessive link. In this learner-goal difference map, there are one excessive links, five lacking links, and four leaving links. By clicking a link label, the text of the origin of the proposition that is used to make the proposition of the link (in the same way shown in Fig. 7) is displayed. By using the text, it is possible to confirm the meaning of the link and the two nodes connected by the link.

### KBmap analyzer: group map

**Fig. 12:**
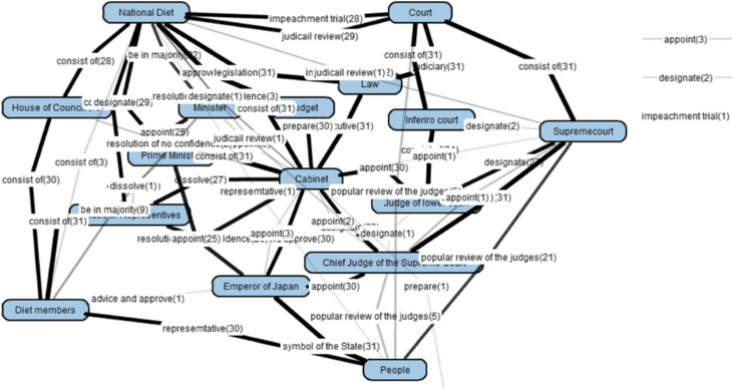
An example of group map

**Fig. 13:**
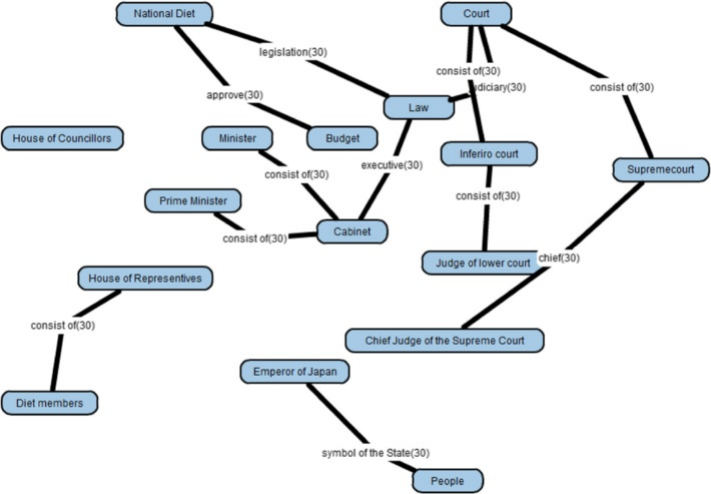
A filtered group map (100 % appearance)

Figure 12 shows a group map generated by the preliminary use reported in the next section. A bold link means that many learner maps include the same connection of the link. By clicking a link label, a list of learners whose learner maps include the link are shown. By using this map, it is possible for a teacher to examine his/her learners’ comprehension. However, because there are a lot of kinds of links in the group map, it is not easy to check it. Therefore, this analyzer has a function to filter links by the ratio of appearance in learner maps. Figure 13 shows a filtered group map composed of only 100 % appearance ratio links, that is, all learner maps include the propositions shown in Fig. 13.

**Fig. 14:**
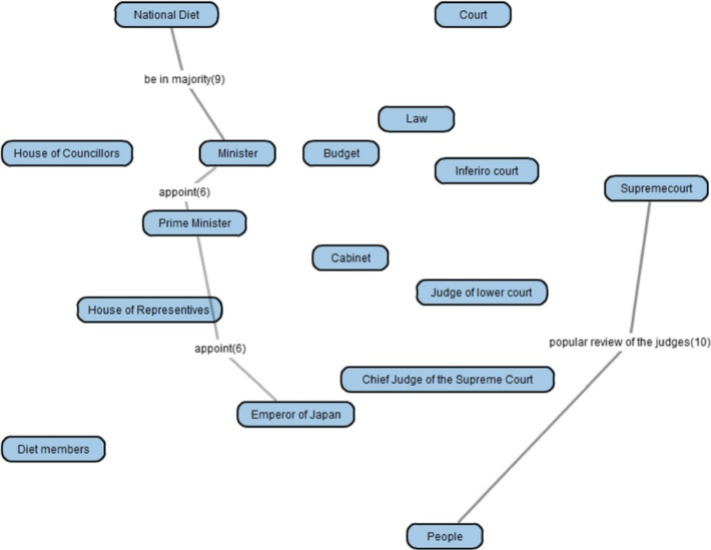
A filtered group-goal difference map (lacking links more than 20 %)

**Fig. 15:**
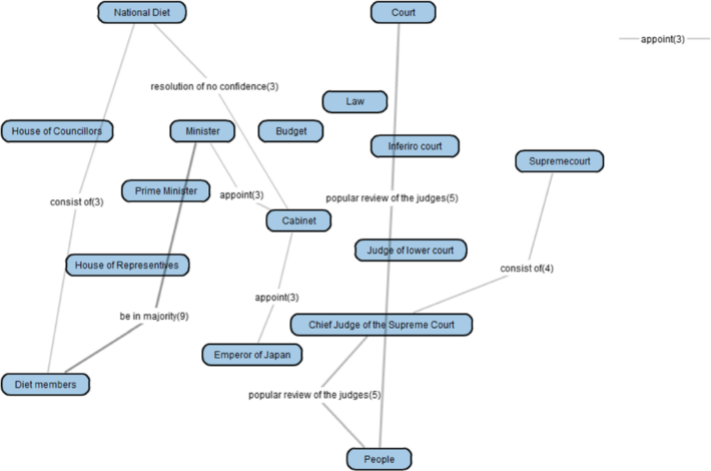
A filtered group-goal difference map (excessive links more than 10 %)

By comparing the group map and the goal map, we can find lacking and excessive links that many learners failed to correctly connect. We call this map “group-goal difference map.” The links in the group-goal difference map can be also filtered. Figure 14 is a filtered group-goal difference map that is composed of lacking links appeared in more than 20 % learner maps. Figure 15 is a filtered group-goal difference map that is composed of excessive links appeared in more than 10 % learner maps.

These different links are basically targets of additional teaching or learning. However, in the framework of kit-build concept map, we suppose that the cause of discrepancy between the goal map and learner map is not only in learner side but also in teacher side as inadequate teaching, learning material, or goal map. Therefore, a teacher is also required to judge whether the different links, that is, excessive, lacking, and leaving links, are acceptable or not by examining related learning material and by reflecting his/her teaching about the topics. If a link is judged acceptable one based on the current teaching or learning material, the goal map is modified to accept the link. The map including such accepted links is called “adjusted goal map.” This modification is not only a method to compensate incompleteness of the goal map but also a promising way to contribute to the improvement of the goal map, learning material, and teaching itself. An example of this modification is explained in the “Results of learner map building activities” section.

### Data structures

**Fig. 16:**
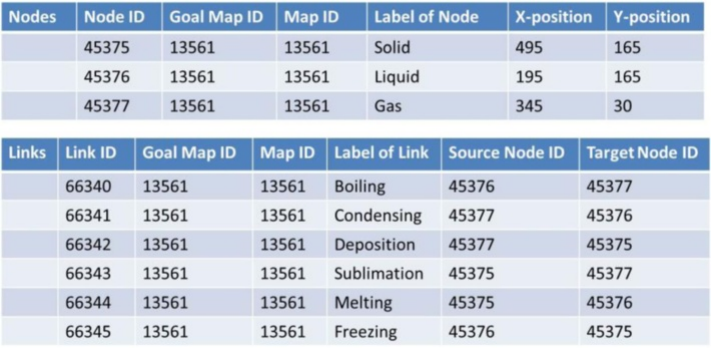
Data of goal map shown in Fig. 2

**Fig. 17:**
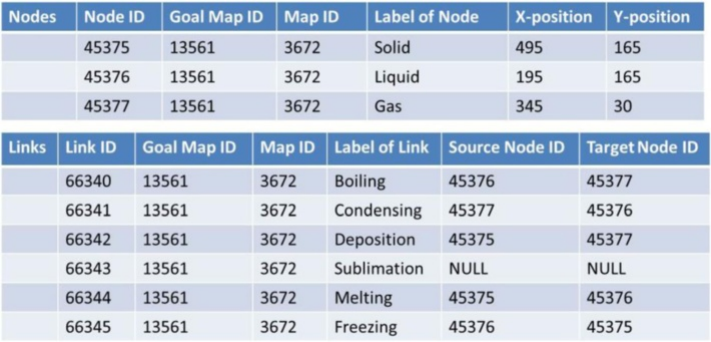
Data of learner map shown in Fig. 4

**Fig. 18:**
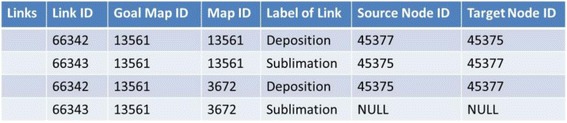
Example of differences between goal map and learner map

**Fig. 19:**
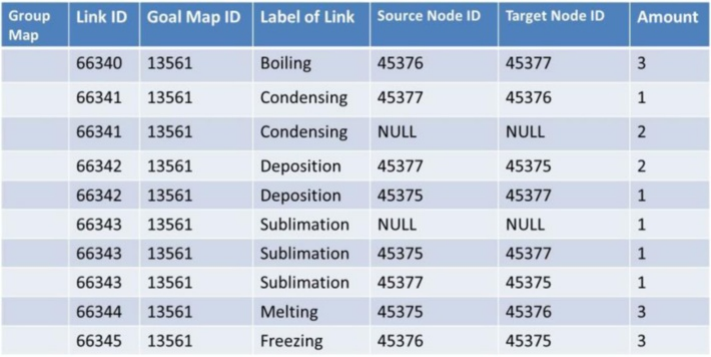
Link data of group map

Figure 16 shows concrete data of nodes and links of the goal map shown in Fig. 2 (only elements related to the diagnosis are displayed). Because all maps are composed of the same nodes and links, there are differences only in the connection of links in the comparison between maps. The differences of the connection are detected by checking the source_node_id and target_node_id. In Fig. 17, data of links of learner map shown in Fig. 4 are shown. By picking up the same id link from two maps and comparing the source_node_id and target_node_id of them, the two maps can be compared. The results of this comparison are shown in Fig. 18. In the phase of a group map building, a pair of nodes connected by every each link is examined in a learner map. The group map is expressed as the total of the examination. Figure 19 shows the data of the group map shown in Fig. 5. A link is able to use to connect every pair of nodes. The link would be often connected to only one node or be connected to no node. In the group map building, as for all links in all learner maps, their connection states are checked and counted. Concrete data of the group map is composed of the set of connection states of the links.

**Fig. 20:**
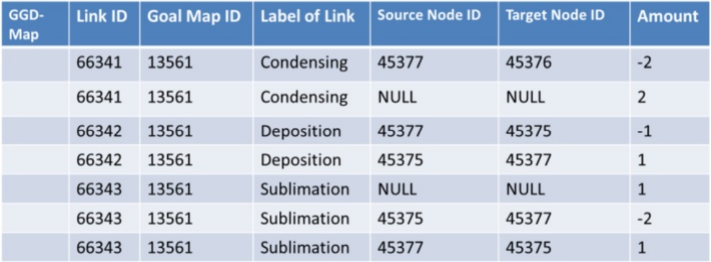
Link data of group-goal difference map

A group-goal difference map is generated by comparing link data of the group map and the goal map. In this comparison, full marks are added to link data of the goal map in additional amount column and then the amount of each link data is compared. Figure 20 is link data of the group-goal difference map shown in Fig. 6. The link data is generated by comparing the link data of goal map shown in Fig. 16 with the link data of group map shown in Fig. 19.

## Preliminary use

### Purpose of preliminary use

In the framework of KB map, we assumed that (1) it is possible for learners to build a map adequately by using a set of parts, (2) the group (including group-goal difference map) map is useful for a teacher to look through the comprehension of the group of learners and to confirm the learning material, and (3) it is possible for the learners to modify their maps following feedback based on the diagnosis. In order to examine whether these assumptions can be realized in the implemented KB map system, we have conducted a preliminary use of the KB map system. We call this “preliminary use” because this is a previous step to use the system in a learning context. In this preliminary use, 30 subjects who were students of undergraduate course of information engineering division attended. The learning material used in this experiment was “separation of powers under the Japanese Constitution.” This topic is a compulsory common knowledge in Japan, and all of the subjects had already learned the topic in their high school periods. Besides, in this experiment, the subjects build their maps while referring to the learning material. In other words, they were not necessary to memorize the contents and were only requested to build their maps as a kind of translation. Although they are not real learners, we thought it was possible to obtain useful suggestion about the above assumption of (1) and (3). Regarding the role of the teacher, we authors took the role. Two of them were in charge of several lectures in a university. Although we were not professional teacher of this learning topic, we thought it was possible to obtain useful suggestion about the above assumption of (2). Because this use is not conducted in a practical learning context, it is indispensable to evaluate the KB map in a real learning context in the next step of this research.

### Procedure of this use

Thirty subjects attended this preliminary use conducted in 2 days. At the first day, the following procedures were carried out. At first, we provided a subject with a learning material about “separation of powers under the Japanese Constitution.” Then, they were requested to read the learning materials in 20 min. Next, we explained “concept map” and “method of making the concept map by the system” in 10 min with several examples. Afterward, the subjects were required to build concept maps of the topic in 20 min with the system. During the phase of the learner map building, the learning material was available in the system. Because the subjects built their maps while reading the learning material, they were not necessary to have special prior knowledge or memorize the contents in the material. They were only requested to build their maps as they understood.

After the learner map building phase, the learner maps were gathered online and analyzed by KBmap Analyzer. At first, by overlaying the learner maps, a group map was generated. By comparing the group map with the goal map, a group-goal difference map was generated. We checked the group-goal difference map and adjusted the goal map. By using the adjusted goal map and each learner map, an individual difference map is made. In the second day, a learner was required to improve their map based on the individual learner map. This map includes leaving, lacking, and excessive links. In this experimental use, because lacking links are the correct answers themselves, only leaving and excessive links were shown to learners and they were requested to improve their maps. Each learner tried to improve his/her maps individually for 20 min. Afterward, the subjects answered the questionnaires.

### Results of learner map building activities

The document of the “separation of powers” is composed of 1101 Japanese characters and six paragraphs. As the goal map building phase, we authors made the goal map composed of 16 nodes and 33 links (Fig. 10). Therefore, a subject was requested to build a learner map with 49 parts (Fig. 8) in the learner map building phase. Thirty learner maps were built by 30 subjects. As for the 30 learner maps, in individual comparison between the goal map and each learner map, KBmap Analyzer detected 63 excessive links (2.1 links in average in a learner map), 69 lacking links (2.3 links in average), and 6 leaving links (0.23 links in average). Because the number of leaving links was only 0.23 links in average, the subjects could use almost parts to build their maps. Then, their maps had usually several defects as excessive links, lacking links, and leaving links. Lacking links can be supplemented by excessive links or learning links. Here, a correction of a lacking link means that either a correction of an excessive link or a correction of leaving link. Therefore, a subject correctly connected 30.7 links (93 % of links) and failed to correctly connect 2.3 links (7 % of links).

As the group map building phase, by overlaying the 30 learner maps, a group map (Fig. 12) was generated. In comparison of the group map and the goal map, KBmap Analyzer detected 61 differences, that is, 26 kinds of excessive links (63 links in total in learner maps), 31 kinds of lacking links (69 in total in learner maps), and 4 kinds of leaving links (6 in total in learner maps). As the goal map modification, we examined all of the detected differences and corresponding portions in the learning material. As the results of the examination, we judged that 4 kinds of excessive links (and corresponding lacking links) should be accepted as alternative interpretations of the learning material. There were two “consist of” links between “National Diet” and “Diet Members” (corresponding correct one in the goal map is “National Diet” and “House of Councillors” or “National Diet” and “House of Representative”) and “Chief of Judge of the Supreme Court” and “Supreme Court” (corresponding correct one is “Court” and “Supreme Court”), one “be in majority” link between “Diet Members” and “Minister” (corresponding correct one is “National Diet” and “Minister”), and one “popular review of the judges” link between “People” and “Chief of the Supreme Court” (corresponding correct one is “People” and “Supreme Court”). In every four cases, although expressions in the learning material were corresponding correct ones, the wrong connections were also acceptable as another interpretation of the expressions. For example, “Supreme Court” is the object of “popular review of the judges” by people as written in the learning material. Then, because “Chief of the Supreme Court” is also a member of “Supreme Court,” he/she is also the object of “popular review of the judges.” Therefore, we judged that the connection between “People” and “Chief of the Supreme Court” with “popular review of the judges” was acceptable. In the same time, it was necessary to connect “People” and “Supreme Court” with “popular review of the judges.” So, we judged that it was inadequate to point out the connections as errors and accepted the four excessive connections as acceptable ones. Moreover, in order to complement the lacking links, we decided to supply the subjects of the four links. Therefore, the adjusted goal map additionally included the four acceptable links and the four complementary links. This modification suggested that it was necessary to improve the corresponding part of the learning material. In this preliminary use, however, we did not discuss the way to revise the learning material itself.

As for this modification phase, we also examined the usefulness of the filtering with the appearance ratio of links in the group-goal difference map. Since 61 differences were too much to check in short time, we tried to filter them by using the overlay degree. When we set the appearance ratio to detect the differences in the group map at 0.1 in the excessive link (that is, 10 % of learner maps included the excess link), 0.8 in the lacking link, and 0.1 in the leaving link, 8 excessive links, 4 lacking links, and 1 leaving link were detected. Then all 4 links that caused the adjustment included were detected. Therefore, the filtering with the appearance ratio might be a useful method to reduce the load to the adjustment.

After this modification, remaining major errors were following 2 excessive links, that is, “popular review of the judges” between “People” and “Court,” and “dissolve” between “Cabinet” and “House of Councilors.” “Popular review of the judges” should be used to connect between “People” and “Supremecourt,” and then, “dissolve” should be used to connect between “Cabinet” and “House of Representatives.” These were written in the learning materials, but a few subjects seemed to fail to distinguish “Supremecourt” and “Court” or “House of Representatives” and “House of Councilors.” These results were also useful to revise the learning materials.

In the phase of learner map modification, each learner is provided with his/her learner-goal difference map that is only composed of excessive and leaving links. Here, because a lacking link in the map indicates a correct link, the lacking links were omitted in the map provided to the learner. The learners were promoted to improve their map for 20 min by referring the provided map learner-goal difference map. Therefore, in the modification phase, the correct answers were not taught directly. In this experiment, 19 in 26 subjects corrected their learner maps completely and 6 subjects left one incorrect link. One subject gave up correcting his map soon and left 5 incorrect links. As the results, 99 % links were correctly connected.

### Results of questionnaires

Table 1 showed the results of the questionnaires that were carried out just after the learner map modification phase. Q1 was a question to ask about total evaluation of the activities in the preliminary use by the subjects. Q2 to Q5 were questions to examine whether building the concept map with a set of parts, that is, kit-building, was acceptable or not for the subjects. Q6 and Q7 were questions to ask about learner map modification.

**Table 1:** Results of the questionnaires

	Questions	Strongly agree	Agree	Disagree	Strongly disagree
(Q1)	Building the concept map was useful to understand the learning material.	17	10	3	0
(Q2)	Kit was helpful to build the concept map.	19	8	3	0
(Q3)	There are enough parts to build the concept map.	4	16	8	1
(Q4)	Building the concept map that represents your understanding was easy.	6	9	15	0
(Q5)	The concept map you built was appropriate.	3	16	10	1
(Q6)	The feedback for the concept map was appropriate.	14	9	1	2
(Q7)	Activity to improve the concept map was useful to understand the learning material.	11	14	1	0

As for Q1, 90 % of the subjects agreed that building the concept map with a set of parts was useful to understand the learning material, and the set of parts were helpful for them to build the concept maps. This result suggests that activities with KB map system were accepted by the subjects as useful activities for learning. Because 90 % subjects agreed that provided kit was helpful to build the concept map. This suggests that the subjects basically accepted to use the kit to build the concept map. However, 30 % subjects did not agree that the kit provided enough parts to build the map although 70 % subjects agreed it. This means that it is necessary to improve the kit. As for Q4 and Q5, about half of them did not agree the questions. Because these responses included the difficulties of the learning materials themselves, we think they are not problems of KB map system. They would suggest that more sophisticated support was necessary to build the concept maps more smoothly. In the answers for Q6 and Q7, 90 % of the subjects answered that the feedback for the concept map was appropriate and the activity to improve the concept map was useful to understand the learning material. These results suggest that learner map modification phase was accepted by the subjects.

### Considerations

In this preliminary use, the subjects correctly connected 93 % links in the first trial. Although answers for Q3, Q4, and Q5 indicated that some of the subjects felt difficulty to complete their maps; in the answers for Q1 and Q2, 90 % of the subjects agreed that to build the concept map was helpful to understand the learning material and also agreed that the kit is helpful to build the map. Based on these results, as for the first assumption, we concluded that most of the subjects were able to build their concept maps adequately by using provided parts in this preliminary use.

By comparing the group map with the goal map, the group-goal difference map was generated. The group-goal difference map showed 61 differences, that is, 26 kinds of excessive links (63 links in the learner maps), 31 kinds of lacking links (69 in the learner maps), and 4 kinds of leaving links (6 links in the learner maps). Then, we examined all of the detected differences and corresponding portions in the learning material. As the results of the examination, we judged that 4 kinds of differences, that is 2 excessive links and 2 lacking links, should be accepted as alternative interpretations of the learning material. These activates could not be carried out without building the group map and the group-goal difference map that are original maps in this framework. Besides, as mentioned in the “Results of learner map building activities” section, all modified links were high-appearance ratio links in the group-goal difference map. This result suggests that the filtering function of detected links was also useful to this modification phase. Therefore, as for the second assumption, we concluded that we could conduct Group Map Building and Modification of Goal Map phase following the framework of KB map.

In the learner map modification phase, the subjects then corrected most of the errors with the feedback. From Q6 and Q7, most of the subjects thought that the feedback for the concept map was appropriate and the activity to improve the concept map was useful to understand the learning material. Besides, most of the subjects thought that building the concept maps with the system was useful for learning from Q1. Based on these results, as for the third assumption, we judged that the subjects were able to modify their maps following to feedback.

We have confirmed that the KB map system can be used along with the framework of the KB map through this preliminary use. Based on this result, we judged that this framework is promising. However, this use was not carried out in a real learning context. So, we have not investigated learning effects of KB map system yet. Therefore, it is indispensable for us to use the KB map system in a real learning context and to analyze the results as the next step of this research. We have already started to use the kit-build concept map in an elementary school in science learning practically (Sugihara, Osada, Nakata, Funaoi, and Hirashima, 2012; Sugihara et al., 2012). Through these practical uses in various learning domain, we will also investigate the generalizability of this framework. The details of these results will be reported in another paper.

## Conclusions

In this paper, we have described the framework of the kit-build concept map as a promising method to realize automatic assessment of a concept map. As an implementation, we have already developed the KB map system and used it as preliminary trial. Through the preliminary use, we have confirmed that it is possible to carry out the four phases of the framework of the KB map. A large-size, long-term, and more practical use of the kit-build concept map is our important next step of this research. Currently, we have started to use the KB map system in an elementary school in science classes. We are also preparing to use the system in a junior high school in geography classes or in an undergraduate course of comprehensive reading of foreign language.

In addition to these practical use of the KB map system, improvement of the phase of goal map building is also important future work. One idea is collaborative authoring of the goal map, and the other is distractors of nodes and links. Collaborative authoring of the goal map will improve the accuracy of the goal map. The distractors will heighten the learning effect of building the concept maps. Through these practical uses and addition of functions, we will develop the KB map system and the framework itself.
